# High turnover renal osteodystrophy due to secondary hyperparathyroidism diagnosed by ^18^F-Fluorocholine combined with ^18^F-NaF PET/CT

**DOI:** 10.1080/0886022X.2021.1918165

**Published:** 2021-05-19

**Authors:** Lin Xiong, Weihua Wu, Yue Chen, Liang Cai, Santao Ou

**Affiliations:** Department of Nephrology, The Affiliated Hospital of Southwest Medical University, Luzhou, Sichuan, China; Sichuan Clinical Research Center for Nephropathy, Luzhou, Sichuan, China; Department of Nuclear Medicine, The Affiliated Hospital of Southwest Medical University, Luzhou, Sichuan, China

Dear Editor,

Renal osteodystrophy (ROD) is a common complication in patients with chronic kidney disease (CKD), especially in hemodialysis patients, which is closely related to the incidence of fracture and mortality. Correctly identifying the type of bone turnover in ROD is essential to guide the formulation of treatment measures. At present, bone biopsy is the gold standard for the diagnosis and classification of ROD, but it is rarely performed clinically. Therefore, nephrologists mainly assess the bone turnover status of ROD by detecting serum parathyroid hormone (PTH) and bone turnover markers. Relatively few papers have reported ROD diagnosed by ^18^F-NaF PET/CT. We report a case of high turnover ROD due to secondary hyperparathyroidism (SHPT) diagnosed by ^18^F-Fluorocholine combined with ^18^F-NaF PET/CT in a patient with maintenance hemodialysis (MHD). As far as we know, this has not been reported previously.

A 48-year-old woman was admitted with dyspnea following the activity of walking fast in 2011. Laboratory examination showed that her serum creatinine level was 1100 μmol/L. As a result, she was diagnosed with chronic renal failure (stage 5 CKD) and began to undergo hemodialysis treatment. With the extension of dialysis time, the concentration of serum PTH gradually increased and maintained at 1000–2000 pg/ml for a long time from 2017 to 2019. She was treated with calcitriol and cinacalcet, but the curative effect was poor. Subsequently, the patient began to complain of lumbago, weakness, stiffness and pain in both lower extremities, and manifested as interstitial claudication.

Considering the high value of ^18^F-Fluorocholine positron emission tomography/computed tomography(PET/CT) examination in tissue imaging of hyperparathyroidism [1], the patient underwent ^18^F-Fluorocholine PET/CT imaging. The maximum intensity projection (MIP) image showed four hyperplastic parathyroid nodules (see the circle in Figure 1(A)). In addition, the axial CT images showed four soft tissue density nodules posterior to the upper and lower poles of the bilateral thyroid lobes, with obvious uptake of choline on the axial PET and PET/CT fusion images(see the arrows in Figure 1(B,D): left superior and right superior; Figure 1(E,G): left inferior and right inferior). Combined with the patient's medical history, the findings on ^18^F-Fluorocholine PET/CT imaging was considered to be SHPT.

**Figure 1.**
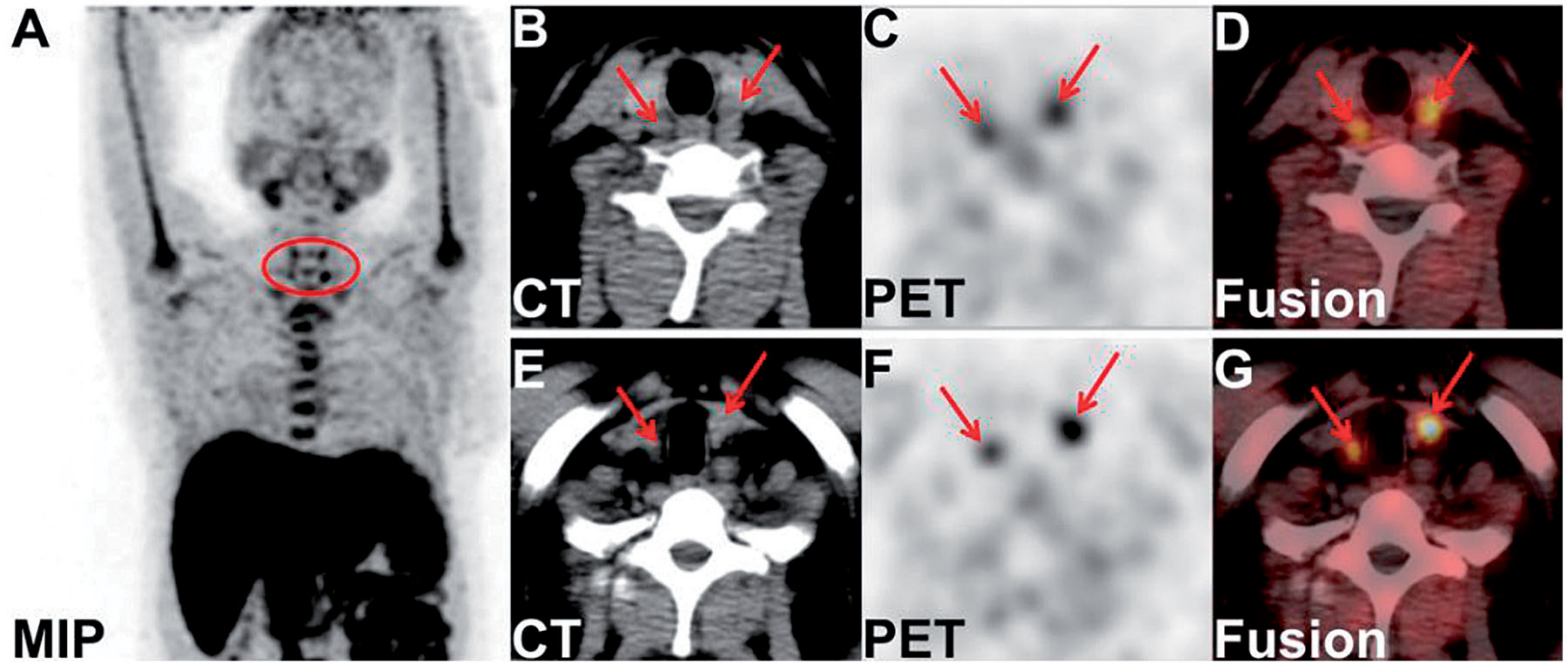
The imaging of parathyroid glands in ^18^F-fluorocholine PET/CT imaging.

Interestingly, besides clearly showing hyperfunctional and hyperplastic parathyroid nodules, the MIP in ^18^F-Fluorocholine PET/CT imaging also revealed significantly increased diffuse choline uptake in the axial bone, pelvis composition bone, bilateral humeri and femurs with some bone changes, considering the possibility of fibrocystic osteitis (Figure 2(A): MIP picture in the anterior plane; Figure 2(B): MIP picture in the posterior plane). To evaluate her bone lesions better, the patient underwent ^18^F-NaF PET/CT whole-body bone imaging. It showed a clear whole-body bone development, extensive uneven bone density with irregular bone structure, and generally increased uptake of radioactive ^18^F-NaF in whole-body bone, of which the concentrated radioactivity in the skull and mandible presented as a ‘black skull sign’, the increased radioactivity uptake in sternum presented as a ‘tie sign’, the symmetrical and punctate radionuclide concentration appears as a ‘beaded rib’ at the junction of the ribs and costal cartilage, and revealed classic ‘super bone imaging’ characterized by obvious development of axial and appendicular skeleton, but blurred visualization of soft tissue and absence of radiotracer excretion in the kidneys and urinary bladder (Figure 2(C): MIP picture in the anterior plane; Figure 2(D): MIP picture in the posterior plane).

**Figure 2.**
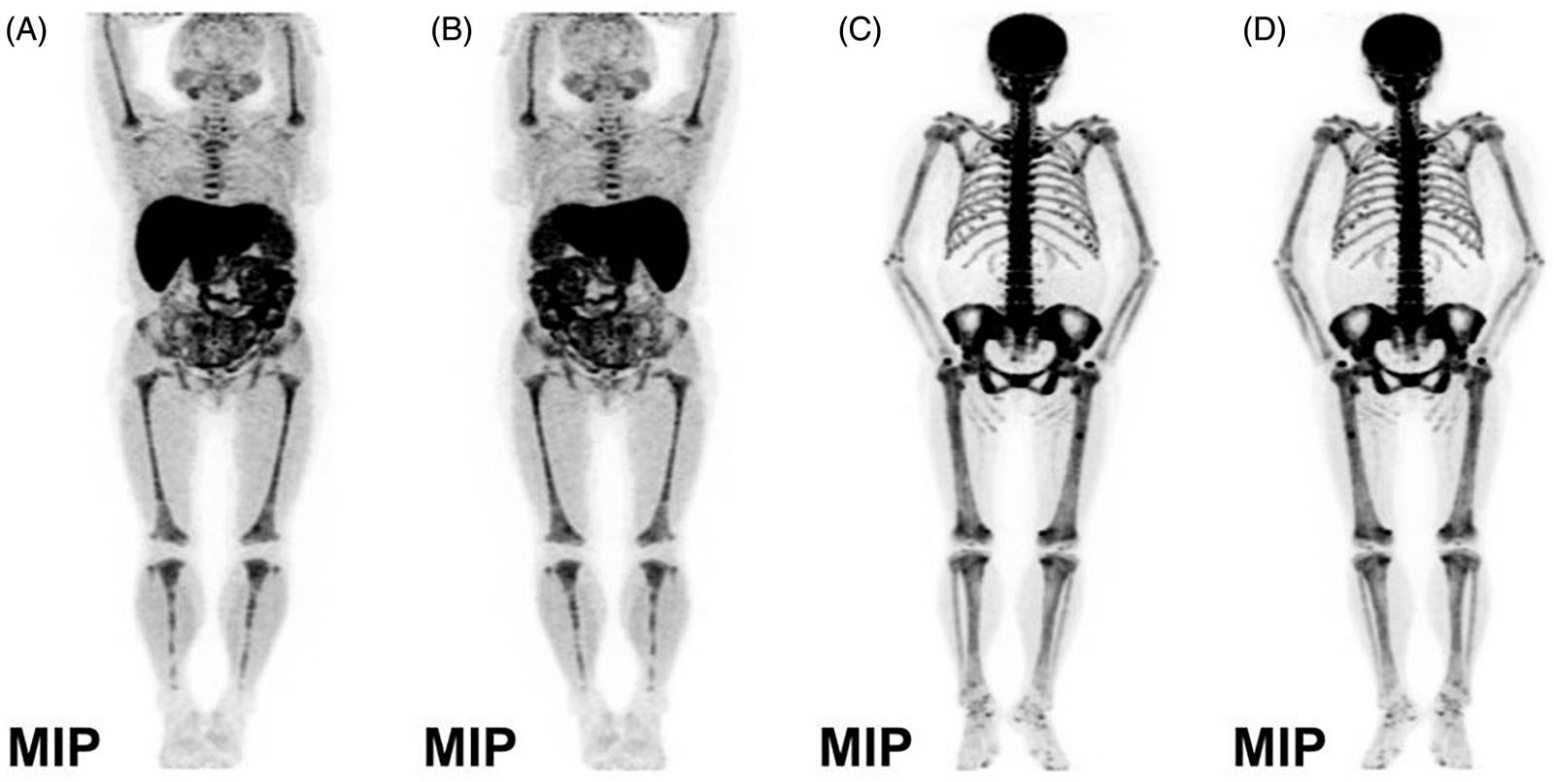
The maximum intensity projection of whole body bone on ^18^F-fluorocholine and ^18^F-NaF PET/CT imaging.

The axial CT and PET as well as PET/CT fusion images of ^18^F-NaF PET/CT whole-body bone imaging showed thickend diploe of cranial bones with increased bone density accompanied by micronodular osteosclerosis in the skull (Figure 3(A–C)); the bone was destroyed at the edges of multiple thoracolumbar vertebral body bones, and the vertebral bodies of T9-L1 had flattened to varying degrees, considering the possibility of pathological fractures(Figure 3(D–F)); the local bone was absorbed and destroyed with uneven bone trabecular density and decreased bone density on the articular surface of the bilateral iliac bones, considering the possibility of fibrocystic osteitis (Figure 3(G–I)). The above imaging features of ^18^F-NaF PET/CT whole-body bone imaging strongly revealed that the MHD patient had active systemic bone metabolism accompanied by partial bone resorption and destruction, which was considered to be a diagnosis of high turnover ROD.

**Figure 3.**
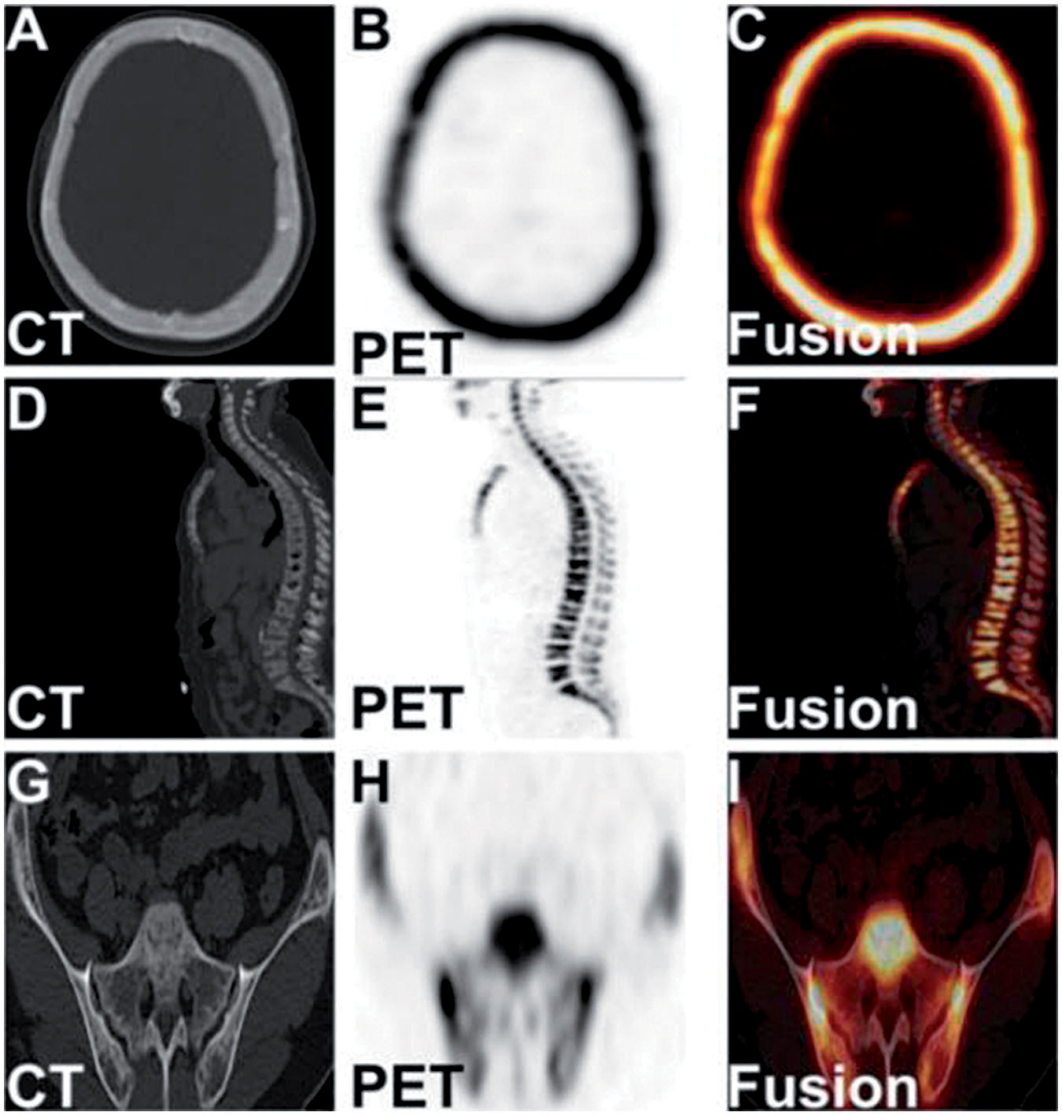
The tomography images of the skull, lumbar spine, and bilateral ilium in ^18^F-NaF PET/CT imaging.

In this case, the imaging characteristics of ^18^F-Fluorocholine PET/CT imaging indicated SHPT, and the long-term fluctuation of serum PTH was between 1000 and 2000 pg/mL and alkaline phosphatase (ALP) was between 400 and 900 U/L(normal range, 35–100 U/L). In addition, laboratory data showed an elevated serum type I collagen β-carboxy-terminal peptide (β-CTX) level of 10 590 pg/ml(normal range, 131–900 pg/ml), and serum type I procollagen amino-terminal peptide (tPINP) level of 1928 ng/ml(normal range, 21.32–112.8 mg/ml), and serum osteocalcin (OC) level of 132 mg/ml(normal range, 8.87–29.05 ng/ml). β-CTX is a marker reflecting bone resorption, and both tPINP and OC are markers reflecting bone formation. These significantly elevated bone turnover markers also indicated an accelerated bone turnover rate. In summary, combining the imaging findings of ^18^F-Fluorocholine PET/CT imaging and ^18^F-NaF PET/CT whole-body bone imaging with significantly elevated serum PTH and bone turnover markers, the MHD patient was clinically diagnosed as high turnover ROD caused by SHPT. Subsequently, the patient was given an intravenous infusion of paricalcitol. Half a year later, the concentration of serum PTH and bone turnover markers decreased significantly, the symptoms of lumbago, weakness, stiffness and pain in lower limbs also disappeared.

## Discussion

ROD refers specifically to the pathological changes in bone associated with CKD, including abnormalities in bone turnover, bone mineralization, bone mass, linear bone growth or bone strength [2], of which the abnormal bone turnover is the main focus on ROD and plays a decisive role in treatment decisions. Bone biopsy is the gold standard for the diagnosis and classification of ROD [2]. Given the invasiveness of the operation and the limited availability of specimen collection and interpretation of results. Clinically, the detection of the levels of serum bone turnover markers, calcium, phosphorus, ALP and PTH as well as the trend of change in serum PTH combined with bone density examination has become a common method for noninvasive diagnosis of ROD [2–3], but the above method are not enough to estimate the classification of bone turnover in ROD. ^18^F-NaF PET/CT whole-body bone imaging can not only assess the overall metabolism of the whole body bone, but also reveal local bone abnormalities. It has the advantage of noninvasive operation, wide examination range, accurate anatomic positioning, high sensitivity and specificity in the diagnosis of bone disease [4–5]. ^18^F-NaF PET/CT imaging of parathyroid adenoma with hyperparathyroidism and osteitis fibrosa cystica has been reported [6]. Moreover, visualization of ROD has been described with the use of ^18^F-Fluorocholine, a tracer for parathyroid imaging [7]. To our knowledge, the simultaneous combination of ^18^F-Fluorocholine PET/CT imaging and ^18^F-NaF PET/CT whole-body bone imaging in the diagnosis of high turnover ROD due to SHPT has not been reported previously.

In conclusion, the present case demonstrates that the combination of ^18^F-Fluorocholine PET/CT imaging and ^18^F-NaF PET/CT whole-body bone imaging combined with other clinical indicators can comprehensively and fully identify the status of ROD, which is expected to be an important mean in the diagnosis of ROD in the future. This is an important subject for nephrologists and warrants further clinical research.

## Data Availability

The datasets used and analyzed during the current study available from the corresponding author on reasonable request.
